# Confinement of ionomer for electrocatalytic CO_2_ reduction reaction via efficient mass transfer pathways

**DOI:** 10.1093/nsr/nwad149

**Published:** 2023-05-23

**Authors:** Xiaowei Du, Peng Zhang, Gong Zhang, Hui Gao, Lili Zhang, Mengmeng Zhang, Tuo Wang, Jinlong Gong

**Affiliations:** School of Chemical Engineering and Technology, Key Laboratory for Green Chemical Technology of the Ministry of Education, Tianjin University, Tianjin300072, China; Collaborative Innovation Center of Chemical Science and Engineering (Tianjin), Tianjin300072, China; School of Chemical Engineering and Technology, Key Laboratory for Green Chemical Technology of the Ministry of Education, Tianjin University, Tianjin300072, China; Collaborative Innovation Center of Chemical Science and Engineering (Tianjin), Tianjin300072, China; National Industry-Education Platform of Energy Storage, Tianjin University, Tianjin300350, China; School of Chemical Engineering and Technology, Key Laboratory for Green Chemical Technology of the Ministry of Education, Tianjin University, Tianjin300072, China; Collaborative Innovation Center of Chemical Science and Engineering (Tianjin), Tianjin300072, China; School of Chemical Engineering and Technology, Key Laboratory for Green Chemical Technology of the Ministry of Education, Tianjin University, Tianjin300072, China; Collaborative Innovation Center of Chemical Science and Engineering (Tianjin), Tianjin300072, China; School of Chemical Engineering and Technology, Key Laboratory for Green Chemical Technology of the Ministry of Education, Tianjin University, Tianjin300072, China; Collaborative Innovation Center of Chemical Science and Engineering (Tianjin), Tianjin300072, China; School of Chemical Engineering and Technology, Key Laboratory for Green Chemical Technology of the Ministry of Education, Tianjin University, Tianjin300072, China; Collaborative Innovation Center of Chemical Science and Engineering (Tianjin), Tianjin300072, China; School of Chemical Engineering and Technology, Key Laboratory for Green Chemical Technology of the Ministry of Education, Tianjin University, Tianjin300072, China; Collaborative Innovation Center of Chemical Science and Engineering (Tianjin), Tianjin300072, China; National Industry-Education Platform of Energy Storage, Tianjin University, Tianjin300350, China; Joint School of the National University of Singapore and Tianjin University, International Campus of Tianjin University, Fuzhou350207, China; School of Chemical Engineering and Technology, Key Laboratory for Green Chemical Technology of the Ministry of Education, Tianjin University, Tianjin300072, China; Collaborative Innovation Center of Chemical Science and Engineering (Tianjin), Tianjin300072, China; Haihe Laboratory of Sustainable Chemical Transformations, Tianjin300192, China; National Industry-Education Platform of Energy Storage, Tianjin University, Tianjin300350, China

**Keywords:** CO_2_ electroreduction, gas diffusion electrode, mass transfer, ionomer, Ag catalyst

## Abstract

Gas diffusion electrodes (GDEs) mediate the transport of reactants, products and electrons for the electrocatalytic CO_2_ reduction reaction (CO_2_RR) in membrane electrode assemblies. The random distribution of ionomer, added by the traditional physical mixing method, in the catalyst layer of GDEs affects the transport of ions and CO_2_. Such a phenomenon results in elevated cell voltage and decaying selectivity at high current densities. This paper describes a pre-confinement method to construct GDEs with homogeneously distributed ionomer, which enhances mass transfer locally at the active centers. The optimized GDE exhibited comparatively low cell voltages and high CO Faradaic efficiencies (FE > 90%) at a wide range of current densities. It can also operate stably for over 220 h with the cell voltage staying almost unchanged. This good performance can be preserved even with diluted CO_2_ feeds, which is essential for pursuing a high single-pass conversion rate. This study provides a new approach to building efficient mass transfer pathways for ions and reactants in GDEs to promote the electrocatalytic CO_2_RR for practical applications.

## Teaser

This paper describes a method of constructing gas diffusion electrodes for electrochemical CO_2_ reduction reaction with uniformly distributed ionomer. The obtained electrodes exhibit a high performance as a result of the enhanced mass transfer of ions and reactant.

## INTRODUCTION

The electrocatalytic CO_2_ reduction reaction (CO_2_RR) is an eminently attractive technology for converting CO_2_ into value-added products using renewable energy (e.g. solar and wind energy) [1–4]. To meet the requirements of industrial applications, it is necessary to develop efficient electrolyzers [5,6]. Membrane electrode assemblies (MEAs) based on gas diffusion electrodes (GDEs) have been widely studied in recent years [7]. The configuration of the MEA not only overcomes the mass transfer limitations in the H-cell but also eliminates the use of cathode electrolyte to mitigate the full cell resistance and improve stability [8,9]. GDEs, as one of the important components in the MEA, are porous electrodes that support catalysts. Generally, a GDE consists of a catalyst layer (CL) and a gas diffusion layer (GDL) [10–12]. The CL is the main place for the catalytic reaction, which normally includes the catalyst to provide the active centers, the catalyst carrier serving as the support, and the ionomer. The ionomer, composed of polymer backbones and hydrophilic groups [13–15], affects the transport of ions and reactants at the catalyst surface, thus impacting the performance of the GDEs [16,17]. Therefore, the ionomer plays an important role during catalytic processes. In order to enhance the electrocatalytic CO_2_RR, the ionomer in the CL should be better homogeneously distributed, which can both prevent catalyst agglomeration and ensure rapid mass transfer [17,18].

Normally, there are two main ways of adding the ionomer to the CL. One is the coating of ionomer onto the CL, which is carried out via ink-free methods (e.g. electrodeposition, sputter and ion-beam deposition approaches) [19–22]. However, the inhomogeneous distribution of the ionomer only on the surface of the CL increases ion transport resistance, leading to deteriorated catalytic performance. The other way of adding the ionomer to the CL is via physical mixing of ionomer into the catalyst ink, which is then coated onto the GDL mainly through drop-casting, hand-painting or spray-coating processes [23,24]. Ink-based approaches are widely adopted due to their advantages of simplicity, flexibility and scalability, which are more suitable for particle catalysts [19,23,25]. However, due to the weak interaction between the catalyst and ionomer in the ink, GDEs obtained by these methods suffer from the random distribution of ionomer. Ionomer aggregation at certain positions in a GDE would lead to high local resistance to mass transfer. At the same time, active centers that are not covered by ionomer might not function properly due to the lack of ion transfer paths [10,26–28]. Thus, effective regulation of the homogeneity of ionomer is essential to promote catalytic performance.

Uniform distribution of ionomer could be achieved by tuning the solvent used in the catalyst ink. Berlinguette *et al.* found that the use of ethanol as ink solvent would facilitate moderate aggregation of ionomer, which in turn optimized its presence in the CL [29]. The obtained GDE possessed promoted selectivity of CO. Improving the interaction between the catalyst carrier and the ionomer can alleviate the aggregation of the ionomer. Strasser *et al.* proposed that the introduction of pyridine/pyrrole functional groups containing N elements on catalyst carriers enhanced their interaction with ionomer. This method promoted the spatial distribution of the ionomer, which improved the efficiency of catalyst utilization and increased the power density of the GDEs [27]. In addition, the optimized allocation of ionomer can be achieved by the modification of ionomer. Yan *et al.* tuned the electrostatic repulsion in the ionomer by optimizing the spacing distance of cationic groups in the ionomer backbone, which regulates the electrostatic repulsion and Van der Waals forces between the ionomer and catalyst. This method prevented the agglomeration of catalyst particles by promoting the distribution of ionomer within the CL, and improved the catalytic activity [17]. Despite the efforts devoted to tuning the presence of ionomer, a more proactive and controllable approach is still needed to further optimize the distribution of ionomer to achieve good performance for the practical application of CO_2_RR systems.

Although there are a lot of works carried out on GDEs modification nowadays, those electrodes made by spraying still suffer from non-uniform distribution of ionomer. Herein, an ionomer pre-confinement method is developed. Specifically, ionomer was introduced during the synthetic process of electrocatalysts, leading to the formation of ionomer-confined electrocatalysts for the construction of GDEs. This method improves the homogeneity of ionomer distribution, building efficient mass transfer pathways. On the one hand, it makes the distribution of pores on the GDEs more average and avoids the high local mass transfer resistance caused by ionomer accumulation, which enhances CO_2_ transport and improves the catalytic performance. On the other hand, it also ensures the successful occurrence of the reaction at the catalytic site and facilitates ion transport within the CL. The promoted CO_2_ mass transfer would lead to a high CO Faradaic efficiency (FE) of over 90% even at a high current density of 600 mA cm^−2^. The enhanced ion transport could result in a decrease in cell voltage (∼3.3 V at 300 mA cm^−2^). In addition, these optimizations also enable the preservation of high selectivity at relatively low CO_2_ concentrations. The optimized electrodes also achieve stable catalysis at a current density of 300 mA cm^−2^ for >220 h.

## RESULTS AND DISCUSSION

### Synthesis and structural characterization of Ag@ionomer and Ag/ionomer catalysts

**Figure 1.**
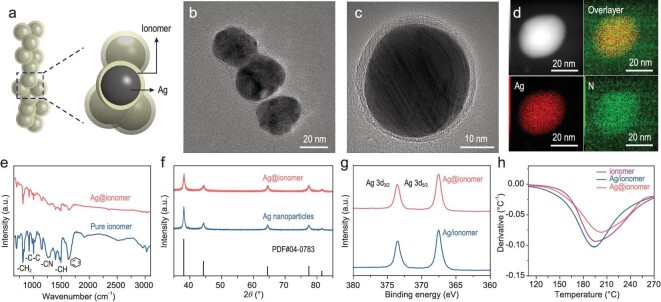
(a) Schematic illustration of the Ag@ionomer. (b and c) TEM images of Ag@ionomer. (d) High-angle annular dark-field scanning transmission electron microscopy image and EDS mapping images of Ag@ionomer. (e) FT-IR spectrum of pure ionomer and Ag@ionomer. (f) XRD patterns of the Ag nanoparticles and Ag@ionomer. (g) XPS spectra of the Ag/ionomer and Ag@ionomer. (h) DTA of ionomer, Ag/ionomer and Ag@ionomer.

Pre-confinement of ionomer was realized by adding PiperION-A5-HCO_3_ anion exchange resin during the synthesis of Ag through the traditional colloidal method (see the Supplementary Data for more details) [30,31]. The electrocatalyst obtained after drying at room temperature is defined as Ag@ionomer (Fig. 1a and Supplementary Fig. 1a). The control sample prepared by physically mixing the ionomer and Ag particles (synthesized by the colloidal method [32], Supplementary Figs 1b and 2) is defined as Ag/ionomer. The Ag@ionomer and Ag/ionomer samples possess similar Ag particle sizes mainly due to the use of the same strong reductant and stabilizer [33,34]. A thin layer of ionomer around the Ag particles can be observed in the transmission electron microscope (TEM) images of Ag@ionomer (Fig. 1b and c). The electron dispersive X-ray spectroscopy (EDS) mappings also demonstrate the uniform distribution of ionomer around the catalyst (Fig. 1d). In addition, the Fourier transformed infrared spectroscopy (FT-IR) spectrum of Ag@ionomer shows peaks consistent with pure ionomer (Fig. 1e), revealing the presence of ionomer in its intrinsic form in the Ag@ionomer. X-ray diffraction (XRD) and X-ray photoelectron spectroscopy (XPS) results indicate that the ionomer does not affect the physicochemical properties of Ag (Fig. 1f and g). Thermogravimetric analysis (TGA) shows that Ag@ionomer and Ag/ionomer possess similar contents of ionomer (i.e. 15 wt%, Supplementary Fig. 3). Differential thermal analysis (DTA) reveals that Ag@ionomer has a higher decomposition temperature than Ag/ionomer and pure ionomer (Fig. 1h). The similar decomposition temperatures of Ag/ionomer and ionomer may be due to the fact that Ag/ionomer is prepared by physical mixing, which leads to only weak interaction by van der Waals force between Ag and ionomer. The higher decomposition temperature indicates the strong interaction between the catalyst and the ionomer in the pre-confined Ag@ionomer electrocatalyst [35,36].

### Fabrication and activity evaluation of GDE_Ag@ionomer_ and GDE_Ag/ionomer_

**Figure 2.**
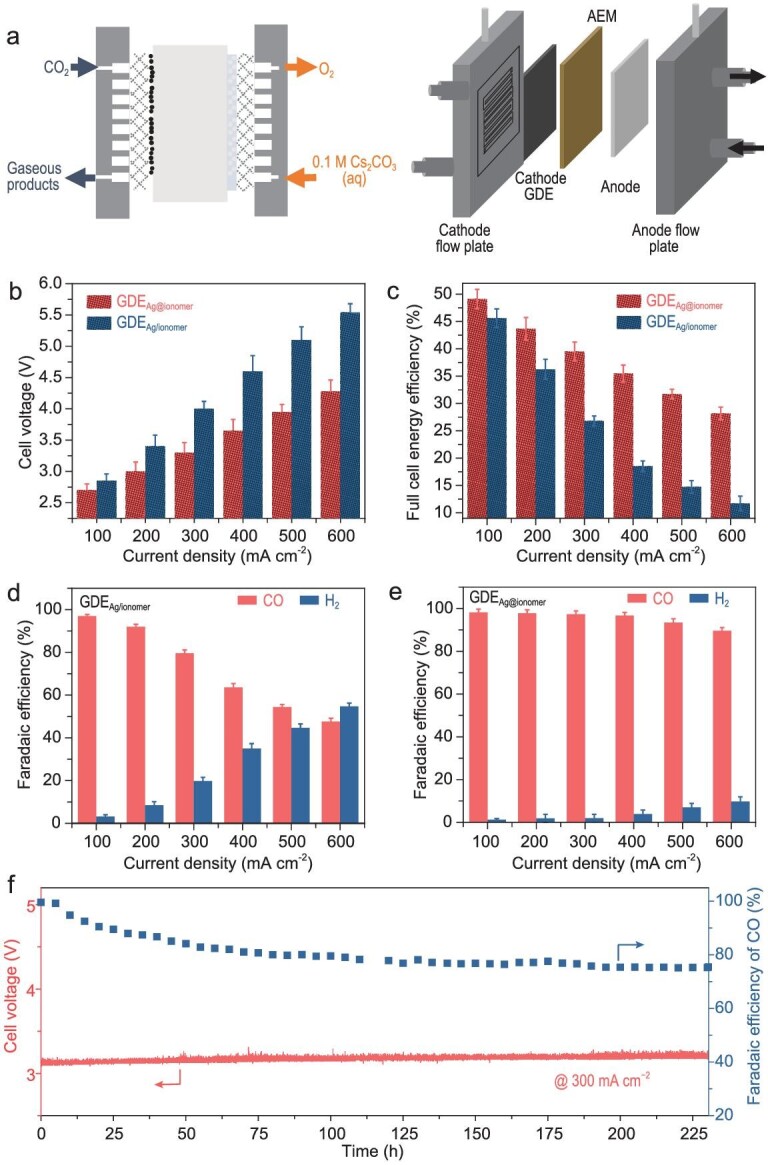
(a) 2D and 3D views of the MEA electrolyzer. (b and c) Cell voltages and full cell energy efficiencies of MEAs with GDE_Ag@ionomer_ and GDE_Ag/ionomer_ as the GDE at different current densities. FEs for CO and H_2_ over (d) GDE_Ag/ionomer_ and (e) GDE_Ag@ionomer_ in the MEA. (f) Stability test of GDE_Ag@ionomer_ at a current density of 300 mA cm^−2^.

**Figure 3.**
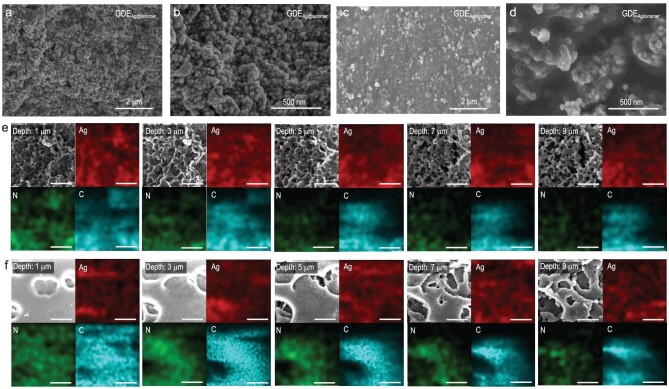
SEM images of (a and b) GDE_Ag@ionomer_ and (c and d) GDE_Ag/ionomer_. FIB-SEM and corresponding EDS mapping images (scale bars: 2 μm) of (e) GDE_Ag@ionomer_ and (f) GDE_Ag/ionomer_.

Subsequently, different GDEs were prepared by airbrushing varied catalyst inks onto carbon GDLs (Supplementary Fig. 4). GDE_Ag@ionomer_ and GDE_Ag/ionomer_ were prepared using the inks with Ag@ionomer and Ag/ionomer dissolved in a mixed solution of isopropyl alcohol and water, respectively (Supplementary Fig. 5). The electrocatalytic performance of the cells was tested in a 4 cm^2^ MEA at room temperature (∼25^o^C) in the galvanostatic mode (Fig. 2a) [37]. The products were analyzed by gas chromatography (GC). According to the results of activity tests for GDE_Ag@ionomer_ and GDE_Ag/ionomer_ with different contents of ionomer (Fig. 2 and Supplementary Figs 6–8), the optimal CO_2_RR performance is achieved when the ionomer content is moderate (∼15 wt%), with the thickness of the ionomer layer on the catalyst surface being ∼1.5 nm (Supplementary Fig. 9). The tests of GDEs with optimized ionomer content indicate that GDE_Ag@ionomer_ exhibits a lower cell voltage and higher energy efficiency than GDE_Ag/ionomer_ (Fig. 2b and c). In addition to the relatively low cell voltage, GDE_Ag@ionomer_ also possesses a higher selectivity towards CO (over 90% even at 600 mA cm^−2^) than the GDE_Ag/ionomer_ (Fig. 2d and e). In order to confirm the source of the products, the composition of CO_2_ feedstock was first checked. The results showed that the feedstock only contained CO_2_ (Supplementary Fig. 10). Subsequently, a control experiment under Ar atmosphere was conducted (Supplementary Fig. 11). When the Ar atmosphere was used, it was found that no carbon-based species were produced. Therefore, it can be determined that CO is generated by the reduction of CO_2_. The stability of the GDEs was tested at a constant current density of 300 mA cm^−2^ (Fig. 2f and Supplementary Fig. 12). In this process, a humidified CO_2_ feedstock with a relative humidity of ∼100% was used, which means that the CO_2_ first passed through a humidification tank containing ultrapure water (at ∼25^o^C) before being fed into the reactor. In the stability test, GDE_Ag@ionomer_ achieved stable operation for >220 h with the FE for CO maintained above 75%. The full cell voltage kept constant at ∼3.2 V with a full cell energy efficiency of ∼32% (Fig. 2f). The CO FE first decreased and then became constant, which is probably due to the water management problem [38–40]. As the reaction proceeds, the generation of salt on the electrodes due to the locally generated OH^−^ makes the electrode progressively hydrophilic (Supplementary Fig. 13). Thus, the electrode becomes over humidified and hydrogen-evolution-reaction (HER) dominated, leading to the decrease of CO FE. Subsequently, the water mass transfer in the reactor reached a balanced state, which in turn maintained the CO FE at a certain level without further decrease. In response to this problem, further investigations (e.g. hydrothermal management of the reaction system, anodic gaseous reactant oxidation) are needed in the future to achieve better system stability. Furthermore, no morphological or structural change was observed for the GDEs after the stability tests (Fig. 3a–d and Supplementary Fig. 14).

To examine whether the prevention of aggregation in Ag@ionomer is the key, an Ag+ionomer sample was prepared by adding ionomer after the reduction of the silver precursor in a typical synthesis of Ag nanoparticles before the centrifugation. In Ag+ionomer, Ag nanoparticles and ionomer are segregated with weak interaction (Supplementary Fig. 15). At the same time, the Ag nanoparticles are well dispersed without aggregation. The performance of GDE_Ag+ionomer_ was far worse than that of the GDE_Ag@ionomer_, which is comparable with that of the GDE_Ag/ionomer_ (Supplementary Fig. 16). These results suggest that the prevention of aggregation in the Ag@ionomer may not be the main reason for its improved performance.

The good performance of GDE_Ag@ionomer_ may be ascribed to the uniform distribution of ionomer as a result of the pre-confinement method. Homogeneously distributed ionomer in the CL may provide more efficient ion transport paths and facilitate the transfer of ions generated at the reaction sites to the anode, which would reduce cell voltage and increase full cell energy efficiency. In addition, the transfer of CO_2_ could be promoted by avoiding local mass transfer resistance caused by ionomer accumulation. This phenomenon would allow easy access for CO_2_ at the active sites and improve the local CO_2_ concentration [41]. CO_2_ surface coverage of the catalyst is directly related to the local CO_2_ concentration, which enhances the selectivity of CO at high current densities.

### Structural characterization of GDE_Ag@ionomer_ and GDE_Ag/ionomer_

In order to verify this hypothesis, we first exclude the possibility that the intrinsic activities of the Ag electrocatalyst in GDE_Ag@ionomer_ and GDE_Ag/ionomer_ are different by Tafel analysis (Supplementary Figs 17 and 18, and Supplementary Table 1) [42,43]. The almost identical exchange current density and Tafel slopes of these two electrodes can provide comparable kinetic data, which indicates that the improvement of the catalytic performance of GDE_Ag@ionomer_ could be attributed to the structural benefits [44–46]. As revealed by a scanning electron microscope (SEM), the GDE_Ag@ionomer_ electrode has a more pronounced porous structure than the GDE_Ag/ionomer_ (Fig. 3a–d), which may be explained by the uniform distribution of ionomer in the GDE_Ag@ionomer_. To better reveal the distribution of ionomer within the CL, focused ion beam SEM (FIB-SEM) characterization with EDS mapping was performed (Fig. 3e and f, and Supplementary Fig. 19) [47]. The results show that GDE_Ag@ionomer_ reserves the porous structure throughout the CL without significant ionomer aggregation, while the ionomer accumulates at the surface of GDE_Ag/ionomer_. The optimized ionomer distribution in GDE_Ag@ionomer_ can also be reflected by the pore size distributions of the electrodes [48]. As revealed by mercury intrusion porosimetry (MIP) experiments (Supplementary Fig. 20) [49], GDE_Ag@ionomer_ has a more concentrated pore size distribution than GDE_Ag/ionomer_. The mutual verification between the results of MIP and FIB-SEM shows that homogeneous distribution of ionomer in GDE_Ag@ionomer_ avoids the blocking of pores caused by the enrichment of ionomer at the surface of GDE_Ag/ionomer_.

### Promoting mechanism of uniformly distributed ionomer in GDE_Ag@ionomer_

**Figure 4.**
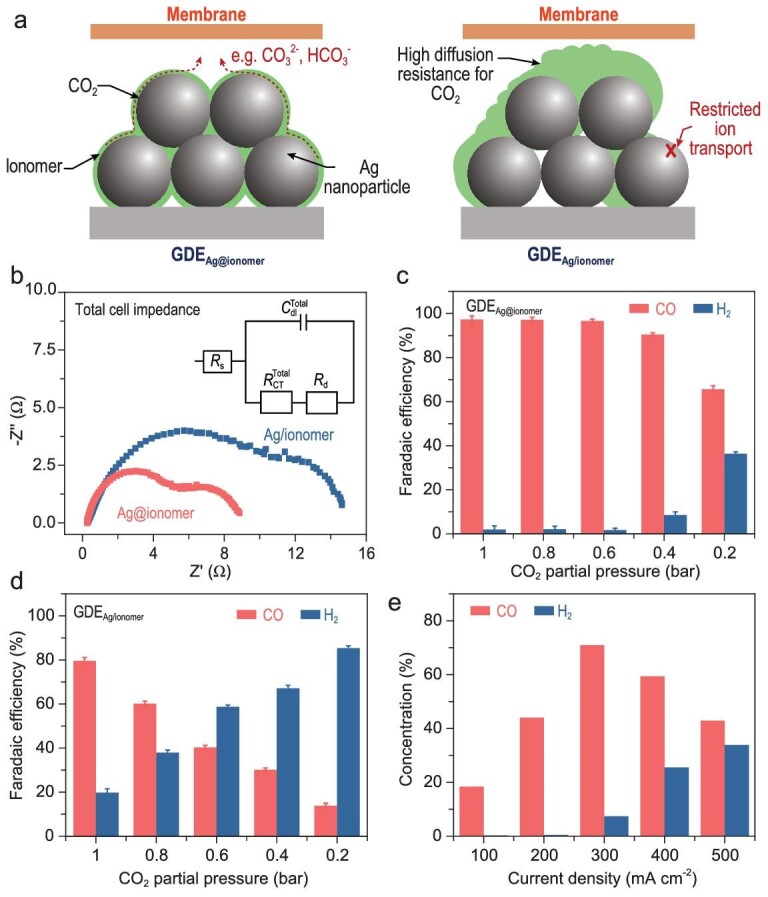
(a) Schematic diagram of the GDE_Ag@ionomer_ and GDE_Ag/ionomer_. (b) Nyquist plots of the impedance related to the electrode. (c and d) Effect of CO_2_ partial pressure on the CO_2_RR performance of GDE_Ag/ionomer_ (c) and GDE_Ag@ionomer_ (d). (e) FEs for CO and H_2_ over GDE_Ag@ionomer_ in the tandem reactor system at different current densities.

The structural characterizations above show that this GDE using the ionomer-confined electrocatalyst optimizes the distribution of ionomer within the CL, building effective ion paths around the catalytic sites (Fig. 4a). To prove that the uniformly distributed ionomer would enhance the ion transport, electrochemical impedance spectroscopy (EIS) tests were performed under operating conditions. The equivalent circuit to which the EIS data were adapted is shown in Fig. 4b, where R_s_ represents internal resistance, ${\mathrm{R}}_{{\mathrm{CT}}}^{{\mathrm{Total}}}$ represents charge transfer resistance, ${\mathrm{C}}_{{\mathrm{dl}}}^{{\mathrm{Total}}}$ represents the sum of cathode and anode electrode capacitance, and R_d_ represents Nernst diffusion impedance [50]. The values of the components in the equivalent circuit are shown in Supplementary Table 2. The high-frequency impedance is obtained by measuring across the entire cell in the two-electrode mode, where the difference is considered to be possibly derived from the cathode part [51]. The results indicate that GDE_Ag@ionomer_ has a lower charge transfer resistance and a lower diffusion impedance than GDE_Ag/ionomer_ (Fig. 4b). With regard to the electron transfer within the GDE_Ag@ionomer_, the negative effect of the ionomer distributed around the catalyst on the electron transfer cannot be denied. However, when there is a thin ionomer layer between catalysts, electrons can still be conducted through a tunneling effect [52,53]. In addition, the catalysts are not completely isolated from each other, which provides pathways for electron transport within the catalyst layer (Fig. 1b). Therefore, the electron transport probably has a minor effect on the successful occurrence of the reaction within the CL. The relatively low diffusion impedance indicates a rapid transfer of reactants and products in the GDE_Ag@ionomer_. Since the ions were generated during the electrochemical CO_2_RR, the well-dispersed ionomer within the CL could facilitate ion transport to reduce the resistance of its diffusion processes [17,51].

In order to prove that the homogeneous distribution of ionomer in the CL can reduce the mass transfer resistance of CO_2_, an activity evaluation using CO_2_ feeds diluted with inert Ar (with CO_2_ concentration down to 20 vol%, see the Supplementary Data for more details) was performed. With a decrease of CO_2_ concentration to 40 vol%, no significant degradation of CO FE was observed for GDE_Ag@ionomer_ at a current density of 300 mA cm^−2^ (Fig. 4c). The CO FE of GDE_Ag@ionomer_ is still ∼70% when the CO_2_ concentration is reduced to 20 vol%. However, the CO FE of GDE_Ag/ionomer_ showed a rapid decreasing trend (Fig. 4d). These results indicate that the homogeneous distribution of ionomer around the catalyst reduces the mass transfer resistance of CO_2_ and improves the utilization efficiency of the active sites. A slight increase in cell voltage can be noticed with a decrease in CO_2_ concentration (Supplementary Fig. 21). For further proof of the enhanced CO_2_ mass transfer, the concentration of CO_2_ within the CL was tracked based on a reaction-diffusion model (Supplementary Fig. 22, see the Supplementary Data for more details). Based on the test results of FIB-SEM and SEM, two porous models with different pore distributions were established to represent the CLs. The GDE_Ag@ionomer_ model exhibits a higher local CO_2_ concentration than GDE_Ag/ionomer_ after providing the models with the same CO_2_ feedstock and current density, which indicates that a more uniform pore distribution facilitates CO_2_ transport within the CL. Therefore, this result further proves that the electrode with uniform distribution of ionomer made by the pre-confinement method has lower mass transfer resistance, promoting the mass transfer of CO_2_ within the CL and increasing the local CO_2_ concentration.

Based on the good performance of GDE_Ag@ionomer_ at low CO_2_ concentrations, a tandem reactor system was built to improve the single-pass conversion of CO_2_. By connecting three MEAs in series, the highest outlet concentration of CO is nearly 71 vol% at a current density of 300 mA cm^−2^ (Fig. 4e). The cell voltages of the three reactors are almost identical at the same current density (Supplementary Fig. 23). To evaluate the universality of this method, another ionomer, i.e. Fumion FAA-3, was examined using this pre-confinement method. During the activity test, the ion exchange membrane was replaced with the matching FAA-3–50. According to the results of activity evaluation (Supplementary Fig. 24), the obtained electrode (GDE_Ag@ionomer (FAA)_) also exhibited promoted activity compared with that prepared by the conventional method (GDE_Ag/ionomer (FAA)_). This pre-confinement method has the potential to be adopted by more systems.

## CONCLUSION

In summary, a pre-confinement method is developed to construct GDEs with homogeneously distributed ionomer in the CL for enhancing mass transfer during the electrochemical CO_2_RR. The uniform ionomer builds paths for the promoted transport of ions, leading to reduced cell voltage, which also facilitates the mass transfer of CO_2_. As a result, easy access to CO_2_ at the active centers would contribute to a high CO FE of over 90% even at a high current density of 600 mA cm^−2^, with the ability to achieve high CO_2_ conversion rates. Moreover, the obtained GDE_Ag@ionomer_ exhibited good stability for more than 220 h at a current density of 300 mA cm^−2^. Considering the high energy conversion efficiency, the high CO FE at high current densities and the good stability, this GDE_Ag@ionomer_ has the potential to realize the electrochemical CO_2_RR in practical applications.

## METHODS

### Materials

AgNO_3_ (99.8%) and NaOH (99%) were purchased from Aladdin Industrial Co. Ltd. Cs_2_CO_3_ (99.9%), isopropyl alcohol (≥99.5%) and ethanol (HPLC, ≥99.8%) were purchased from Macklin Biochemical Co. Ltd. Sodium citrate anhydrous (99%) was purchased from J&K Scientific Ltd. L (+) − Ascorbic acid (≥99.7%) was purchased from Tianjin Kemiou Chemical Reagent Co. Ltd. All chemical reagents were utilized without further purification. Commercially available carbon-based GDLs (AvCarb GDS3250) were purchased from Xima Laya Photo-Electric Technology Co. Ltd., China. PiperION-A5-HCO_3_ anion exchange resin, Fumion FAA anion exchange resin, PiperION-A15-HCO_3_ and FAA-3-50 were purchased from SCI Materials Hub. CO_2_, N_2_, Ar and H_2_ were all purchased from Air Liquide (≥99.999%). The ultrapure water (18.25 MΩ·cm) was supplied by a Millipore Direct-Q5 UV water purification system.

### Catalyst synthesis

Ag nanoparticles were synthesized by using L (+) − Ascorbic acid as the reductant and sodium citrate anhydrous as the stabilizer [32]. Ag@ionomer nanoparticles were prepared under the same conditions. The difference was that 0.045 g of PiperION anion exchange resin was first dissolved in 30 mL of ethanol and mixed with the AgNO_3_ solution before being added to the reducing agent solution.

### Fabrication of electrodes

For the preparation of electrodes, 65 mg of Ag nanoparticles with 0.23 g of 5 wt% ionomer solution were dispersed in 4 mL of isopropyl alcohol and 4 mL of water to form Ag catalyst ink. After 1 h of sonication, the catalyst ink was sprayed onto 25 cm^2^ carbon paper to fabricate GDE_Ag/ionomer_. GDE_Ag@ionomer_ was also prepared in a similar manner with the catalyst ink consisting of 65 mg of Ag@ionomer catalyst, 4 mL of isopropyl alcohol and 4 mL of water. During the preparation of GDEs, a hotplate and infrared lamp were used to accelerate the evaporation of the solvent.

### CO_2_RR performance test in the MEA

The homemade 4 cm^2^ MEA (Gaossunion Co. Ltd.) consists of a GDE, an anion exchange membrane (PiperION-A15-HCO_3_, SCI Materials Hub) and an IrRu/Ti anode. The PiperION ion-exchange membrane (15 μm, without polytetrafluoroethylene (PTFE)-reinforced) was mounted between a cathode GDE and an IrRu-coated Ti mesh anode, with the CL of the GDE oriented towards the membrane [35]. During the testing process, 0.1 M Cs_2_CO_3_ solution served as the anolyte and the humidified CO_2_ flow (relative humidity ∼100%), controlled by the mass flow meter, was supplied to the cathode which had an inlet flow rate controlled at 50 sccm. Since CO_2_ at the cathode can react with OH^−^ to form HCO_3_^−^/CO_3_^2−^, another mass flow meter was used to detect the outlet gas flow rate in order to ensure the accuracy of the gas product selectivity calculation. A fresh GDE, IrRu/Ti anode and anion exchange membrane were used for each electrocatalytic test.

### Characterization

The phase structures were characterized by XRD (Bruker, D8-Focus, Cu Kα radiation) at 40 kV and 40 mA. SEM micrographs were acquired using a Hitachi S-4800 focused ion beam SEM with an accelerating voltage of 5 kV. TEM images were taken on a JEOL JEM-2100F operated at an acceleration voltage of 200 kV. XPS measurements were performed on a Physical Electronics PHI 1600 ESCA system with an excitation source of Al Kα = 1486.6 eV.

## Supplementary Material

nwad149_Supplemental_File
