# Supplementary material for: Confinement of ionomer for electrocatalytic CO2 reduction reaction via efficient mass transfer pathways
Source: Natl Sci Rev. 2023 May 23;11(2):nwad149. doi: 10.1093/nsr/nwad149 (PMC10776366; doi:10.1093/nsr/nwad149)
Supplement: nwad149_Supplemental_File [file nwad149_supplemental_file.pdf]

Supporting Information

Confinement of Ionomers for Electrocatalytic CO_2 Reduction Reaction *via* Efficient Mass Transfer Pathways

Xiaowei Du[1,2,#], Peng Zhang[1,2,4,#], Gong Zhang[1,2], Hui Gao[1,2], Lili Zhang[1,2], Mengmeng Zhang[1,2], Tuo Wang[1,2,4,5] and Jinlong Gong[1,2,3,4,*]

[1]School of Chemical Engineering and Technology; Key Laboratory for Green Chemical Technology of Ministry of Education, Tianjin University, Tianjin 300072, China;

[2]Collaborative Innovation Center of Chemical Science and Engineering (Tianjin), Tianjin 300072, China;

[3]Haihe Laboratory of Sustainable Chemical Transformations, Tianjin 300192, China;

[4]National Industry-Education Platform of Energy Storage, Tianjin University, Tianjin300350, China;

[5]Joint School of National University of Singapore and Tianjin University, International Campus of Tianjin University, Fuzhou 350207, China

*Corresponding author. E-mail: jlgong@tju.edu.cn.

#Equally contributed to this work.

Experimental Section

Catalyst synthesis

Ag nanoparticles were synthesized by using L (+) − Ascorbic acid as the reductant and sodium citrate anhydrous as stabilizer [1]. Typically, a 200 mL aqueous solution containing 0.23 g ascorbic acid and 0.77 g sodium citrate anhydrous was adjusted to reach pH 11 by the addition of 0.1 mol L^{-1} NaOH solution. Subsequently, 4 mL of 0.5 mol L^{-1} aqueous solution of $AgNO_3$ was added in the above aqueous solution. In this process, the solution was placed in a 30 ℃ water bath and with a stirring at the speed of 900 rpm [1]. The reaction solution changes from colorless to black instantly. After 150 min, Ag nanoparticles were obtained by centrifugation at 9000 rpm for 5 min. Ag@ionomer nanoparticles were prepared under the same conditions. The difference is that 0.045 g of PiperION anion exchange resin was first dissolved in 30 mL of ethanol and mixed the $AgNO_3$ solution before being added to the reducing agent solution. Subsequently, Ag@ionomer catalyst was obtained by centrifugation. Ag+ionomer was synthesized by adding ionomer after the reduction of the silver precursor and before centrifugation. All catalysts were dried at room temperature.

CO_2RR performance test in a tandem reactor

In order to improve the single-pass conversion of CO_2, a tandem reactor system was built. In this system the cathodes of three MEAs were connected in series, where each MEA was assembled under the same conditions. In this case, three peristaltic pumps are used to deliver electrolyte to each MEA anode separately (0.1 M Cs_2CO_3 solution was served as the anolyte).

CO_2RR performance test with different CO_2 partial pressure

After the MEAs were assembled under the same conditions, the partial pressure experiment was performed with CO_2 concentration vary from 100 vol% to 20 vol%. The CO_2 concentration was controlled by using two mass flowmeters and a triple valve before electrolysis with a mixture of CO_2 and Ar. The total gas flow rate was ensured to be constant at 50 sccm during the process.

Tafel analysis

The Tafel analysis were performed in a homemade three-electrode MEA reactor (Supplementary Fig. 17) [2]. The Tafel curves of the two samples were obtained by getting the partial current density of CO (j_{CO}) at different overpotentials and subsequently plotting the overpotential (η) versus Log (j_{CO}).

Analysis of CO_2RR products

The gaseous products (CO, H_2) from the outlet of the electrolyzer were delivered to the gas chromatography (GC, Shimadzu, GC-2014) for online analysis after introduced into a gas-liquid separation trap. After 300 s of electrocatalytic process, a GC analysis program run was initiated. A flame ionization detector (FID) equipped with a methanizer was used to detect CO. H_2 was quantified by a thermal conductivity detector (TCD).

Electrochemical impedance spectroscopy (EIS) test

The EIS test was conducted for each zero-gap cell when it operated at a given voltage with a modulation frequency from 100 kHz to 0.1 Hz [2, 3]. The Nova 2.1 (Autolab PGSTAT204, Metrohm) program was used to fit the collected EIS data. The equivalent circuit was used to fit these EIS data specifically, where R_s, R_{CT} and C_{dl} represent the internal impedance, charge transfer resistance and electrode capacitance, respectively (inserted in Fig. 4b). To ensure the reasonableness of the fit, it was made sure that the fitted Chi-Square test value of each sample was less than 0.05.

Calculation of the faradaic efficiency (FE)

FE is calculated as follows:

$$FE_x = \frac{n_x \times n_e \times F}{Q} \times 100\%$$

Where n_x represents the amount of the product x (mol), n_e is the number of electrons transferred in the generated product x, F is the faraday constant (96485 C mol^{-1}) and Q is total number of electrons transferred during the reaction [4].

Calculation of the full cell energy efficiency (EE)

EE is calculated as follows:

$$EE_x = \frac{E_{thermodynamic}}{E_{cell\ voltage}} \times FE_x \times 100\%$$

Where $E_{thermodynamic}$ is the thermodynamic potential of the product x generated during the electroreduction of CO_2, $E_{cell\ voltage}$ is the voltage across the reactor during the reaction and FE_x is the faradaic efficiency of product x [4].

Mass transport simulations

The concentration of CO_2 within the catalyst layer was tracked based on a reaction-diffusion model. A cuboid with a height of about 10 μm was selected as the calculation model. And two porous media models were established based on FIB-SEM and SEM test results. In the $GDE_{Ag/ionomer}$, there is a clear difference in pore size between the electrode surface and the inside of the catalyst layer, while $GDE_{Ag@ionomer}$ possesses a similar pore size structure throughout the catalyst layer (Fig. 3). Therefore, $GDE_{Ag@ionomer}$ was set up as a model with uniform pore size, while the model of $GDE_{Ag/ionomer}$ was reduced to a gradual increase of pore size from the electrode surface to the inner catalyst layer. In the model, a constant supply of CO_2 feedstock was set up at the back of the GDEs and the transport of CO_2 in the models was dominated by diffusion. The diffusive flux is calculated using a mixture averaged diffusion model [5]:

$$j_i = -\rho_g D_i^{eff} \nabla \omega_i - \rho_g D_i^{eff} \omega_i \frac{\nabla M_n}{M_n}$$

where ω_i is the mass fraction of species i, ρ_g is the gaseous mixture density, M_n is the average molar mass of the mixture ($M_n = (\sum_i \frac{\omega_i}{M_i})^{-1}$) and D_i^{eff} is the effective diffusion coefficient for species i. In addition to CO_2 diffusion, the same current density is used as the source of CO_2 consumption in the models.

Results and Discussion

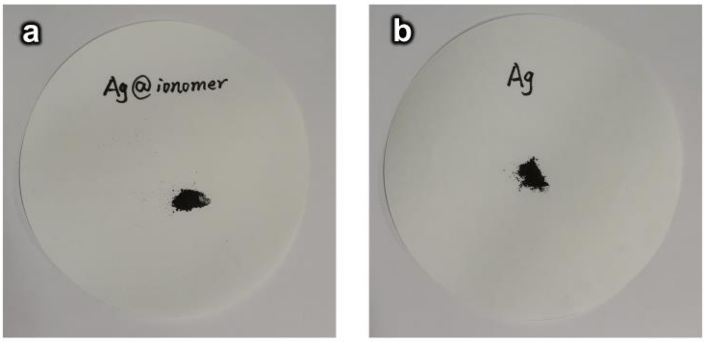

Supplementary Figure 1. Photos of the (a) Ag@ionomer and (b) Ag samples after drying at room temperature.

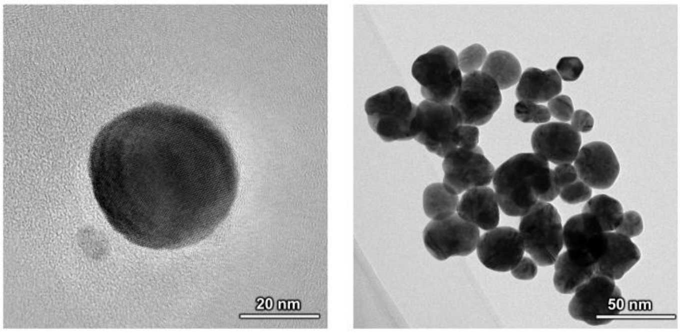

Supplementary Figure 2. TEM images of Ag nanoparticles.

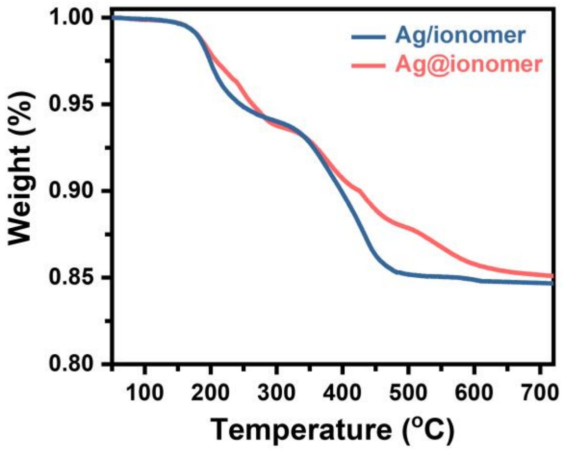

Supplementary Figure 3. TGA of Ag@ionomer (15 wt% ionomer content) and Ag/ionomer (15 wt% ionomer content).

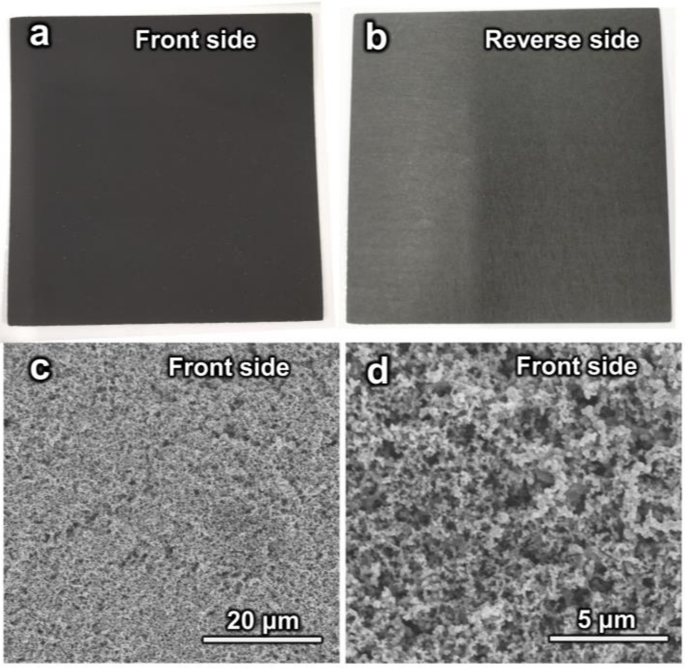

Supplementary Figure 4. (a, b) Photograph and (c, d) SEM images of the GDL at different scales.

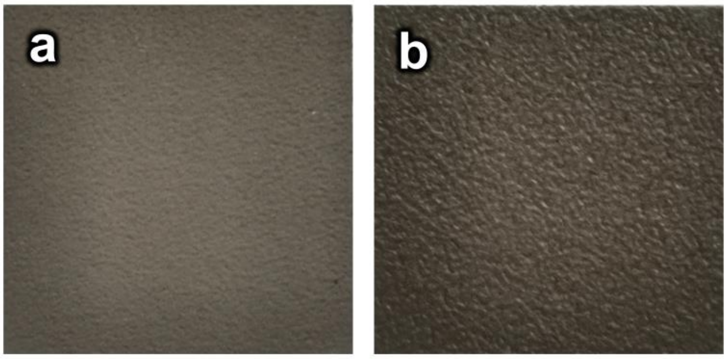

Supplementary Figure 5. Photograph of (a) $GDE_{Ag@ionomer}$ and (b) $GDE_{Ag/ionomer}$.

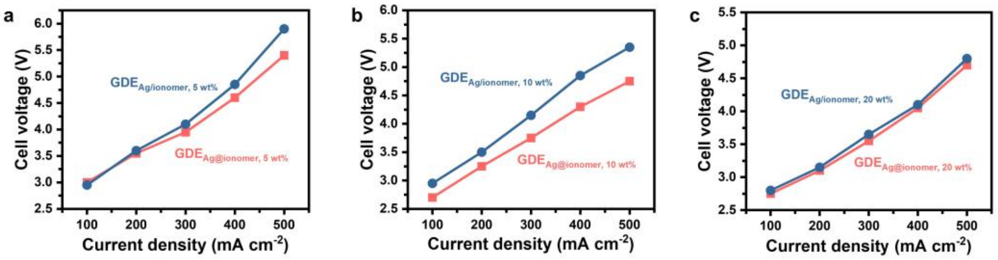

Supplementary Figure 6. Voltages of cells assembled by $GDE_{Ag/ionomer}$ and $GDE_{Ag@ionomer}$ with (a) 5 wt%, (b) 10 wt% and (c) 20 wt% of ionomers at different current densities.

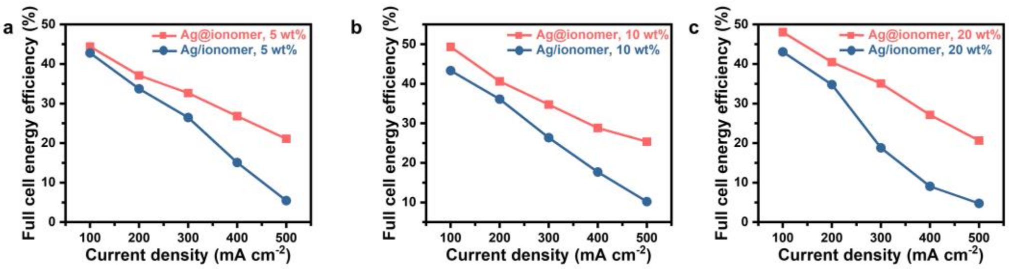

Supplementary Figure 7. Energy efficiencies of cells assembled by $GDE_{Ag/ionomer}$ and $GDE_{Ag@ionomer}$ with (a) 5 wt%, (b) 10 wt% and (c) 20 wt% of ionomers at different current densities.

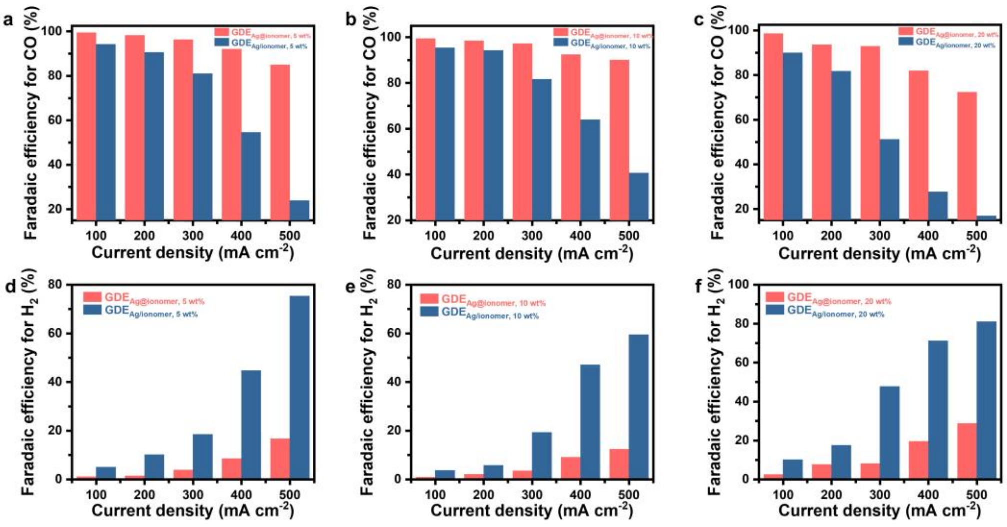

Supplementary Figure 8. FE for CO and H_2 over $GDE_{Ag/ionomer}$ and $GDE_{Ag@ionomer}$ with (a, d) 5 wt%, (b, e) 10 wt% and (c, f) 20 wt% of ionomers at different current densities.

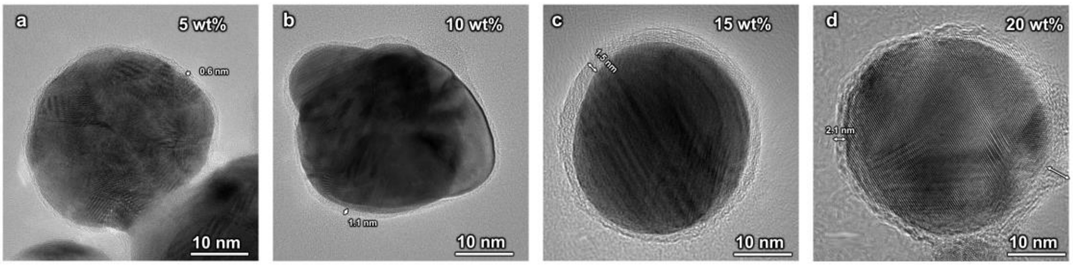

Supplementary Figure 9. Thickness of ionomer layer on the surface of Ag@inonomer electrocatalysts with different ionomer contents.

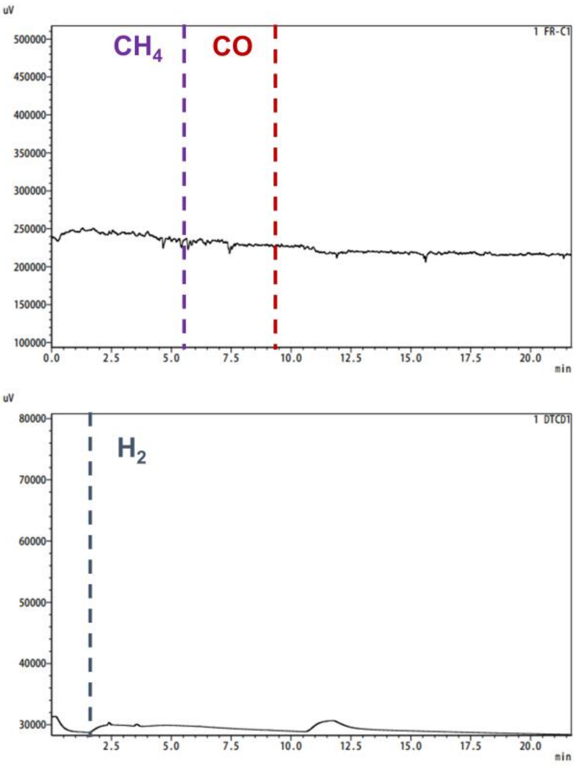

Supplementary Figure 10. Chromatographic profiles of CO_2 feedstock.

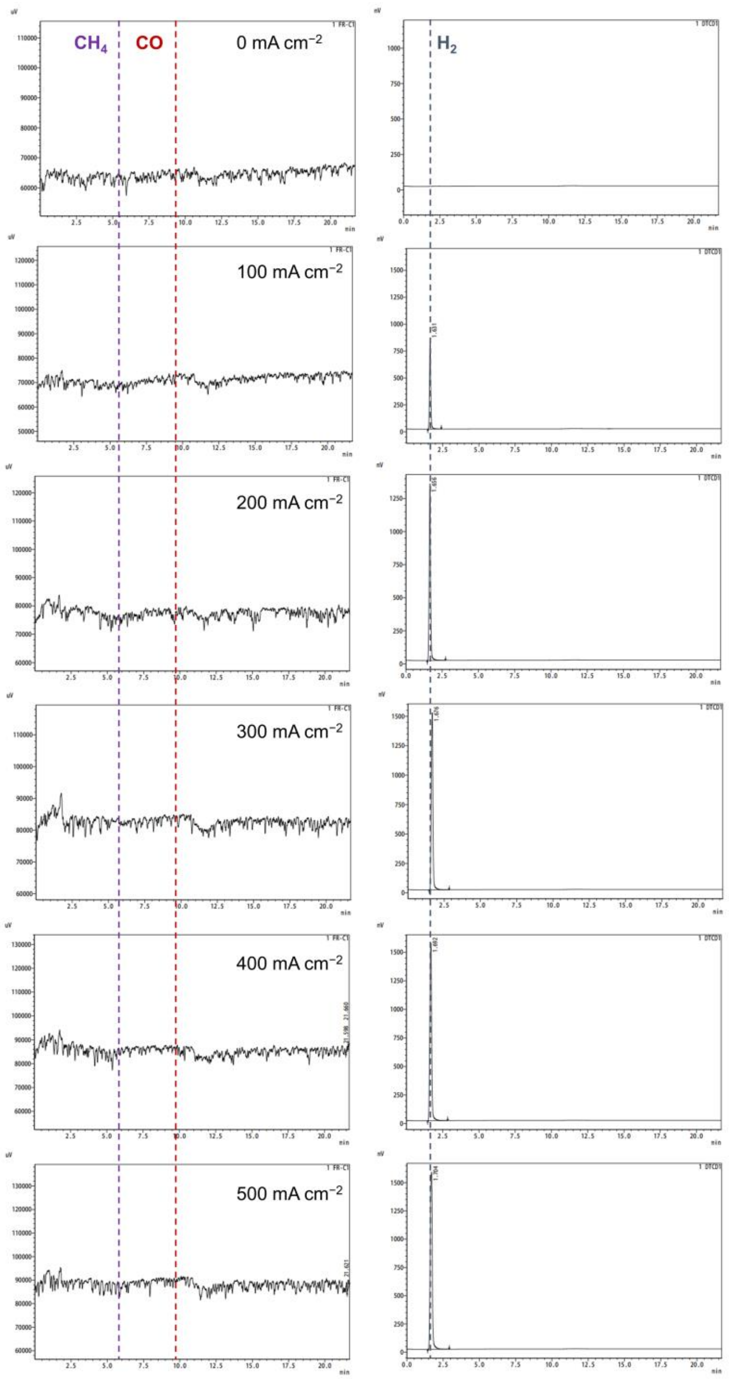

Supplementary Figure 11. The products distribution on $GDE_{Ag@ionomer}$ under Ar gas flow.

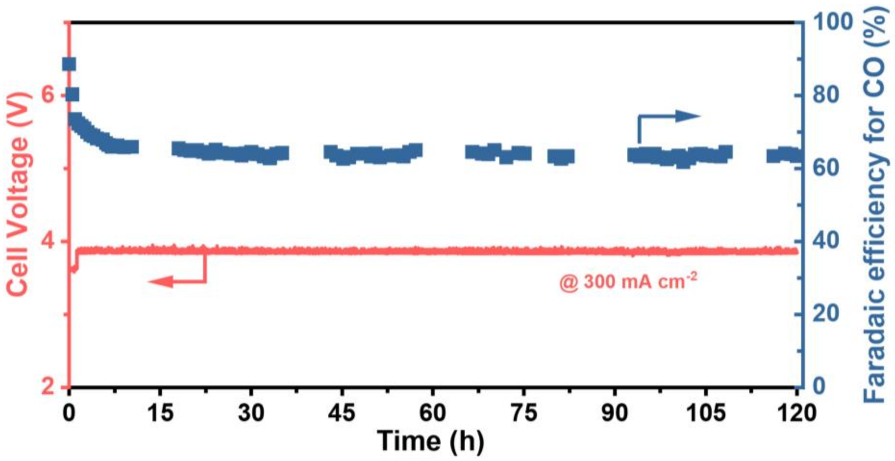

Supplementary Figure 12. Stability test of $GDE_{Ag/ionomer}$ at the current density of 300 mA cm^{-2}.

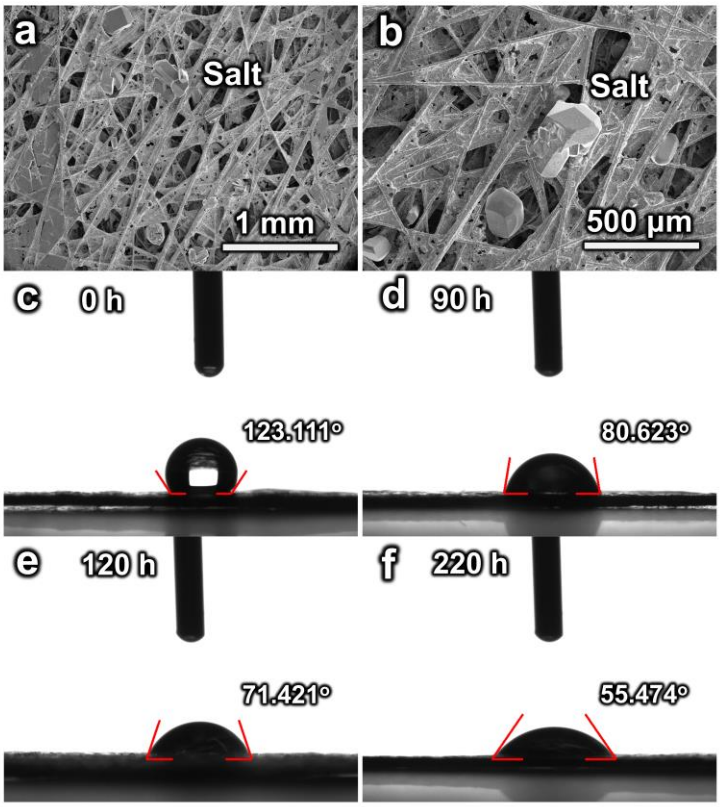

Supplementary Figure 13. (a, b) SEM images of the salt on the reverse side of electrode. Contact angle after different test times (c) 0 h, (d) 90 h, (e) 120 h and (f) 220 h.

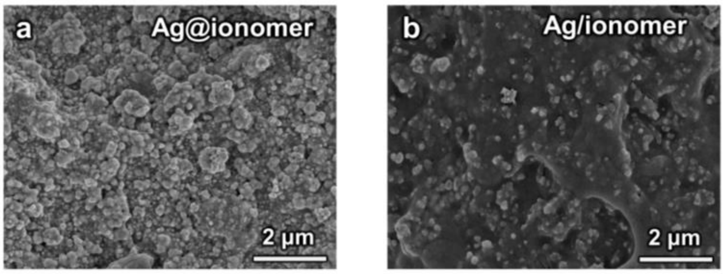

Supplementary Figure 14. SEM images of (a) $GDE_{Ag@ionomer}$ and (b) $GDE_{Ag/ionomer}$ after CO_2RR stability test.

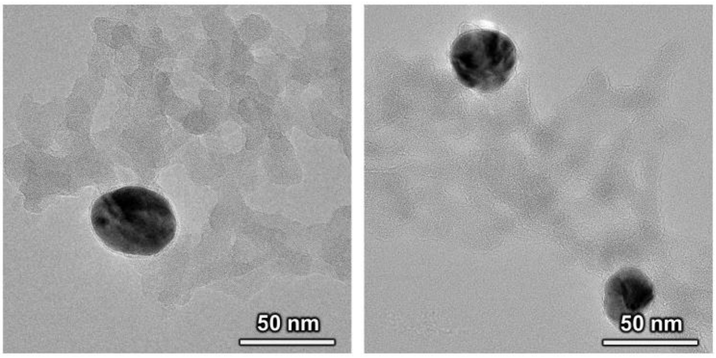

Supplementary Figure 15. TEM images of Ag+ionomer.

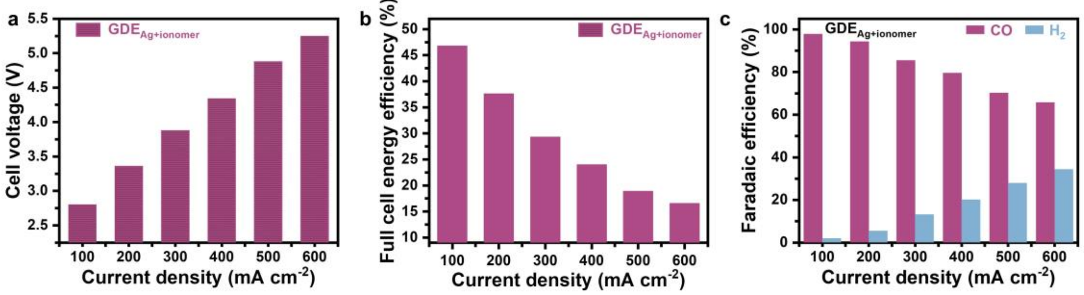

Supplementary Figure 16. (a) Cell voltages, (b) full cell energy efficiency and (c) FEs for CO and H_2 of MEA with $GDE_{Ag+ionomer}$ as the GDE at different current densities.

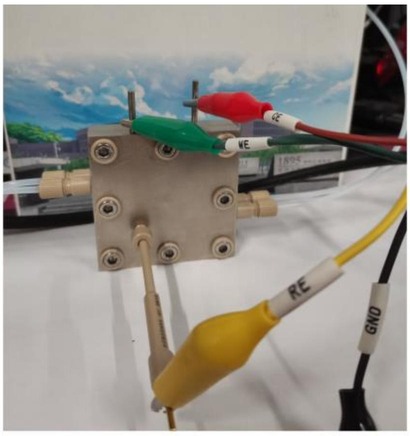

Supplementary Figure 17. Homemade three-electrode MEA reactor.

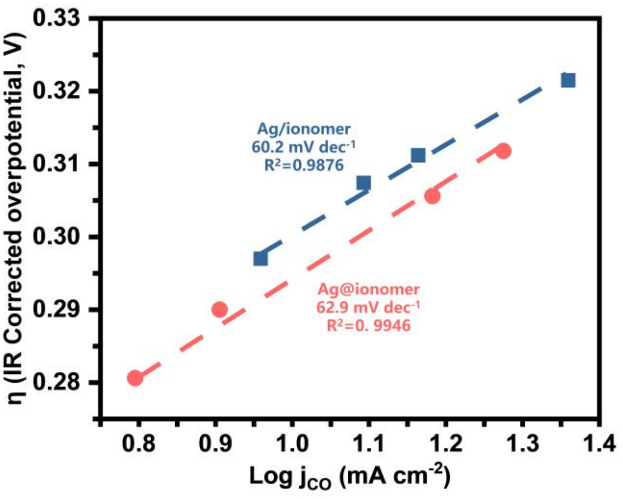

Supplementary Figure 18. Tafel plots of the Ag/ionomer and Ag@ionomer.

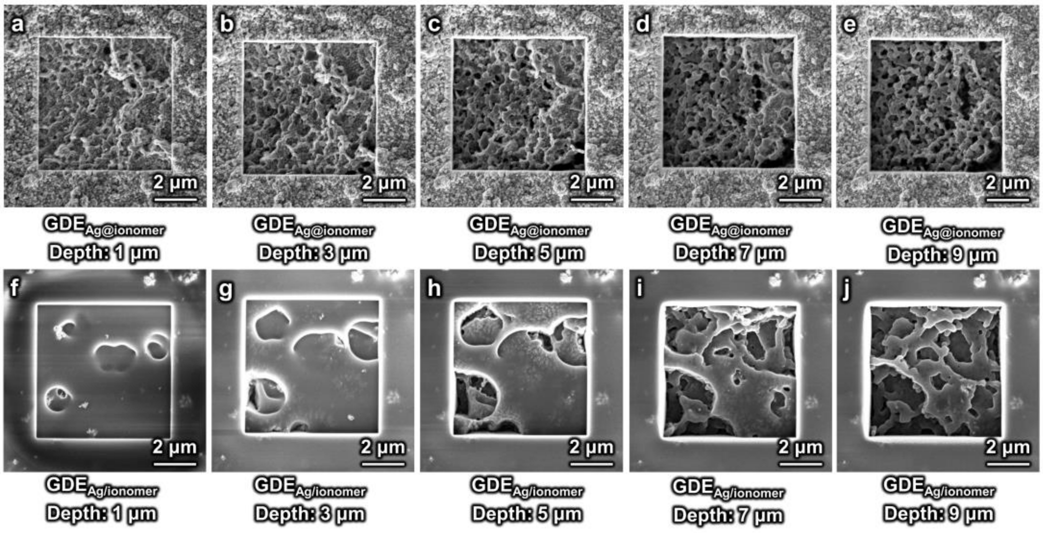

Supplementary Figure 19. FIB-SEM images of (a-e) $GDE_{Ag@ionomer}$ and (f-j) $GDE_{Ag/ionomer}$.

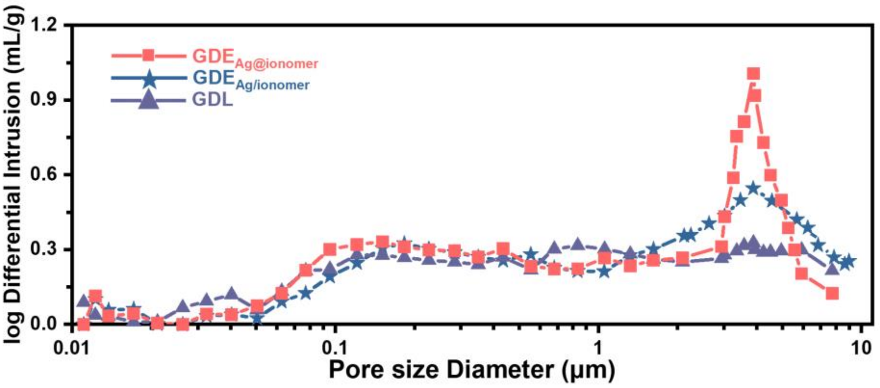

Supplementary Figure 20. Pore size distributions of gas diffusion layer (GDL) and gas diffusion electrodes (GDEs).

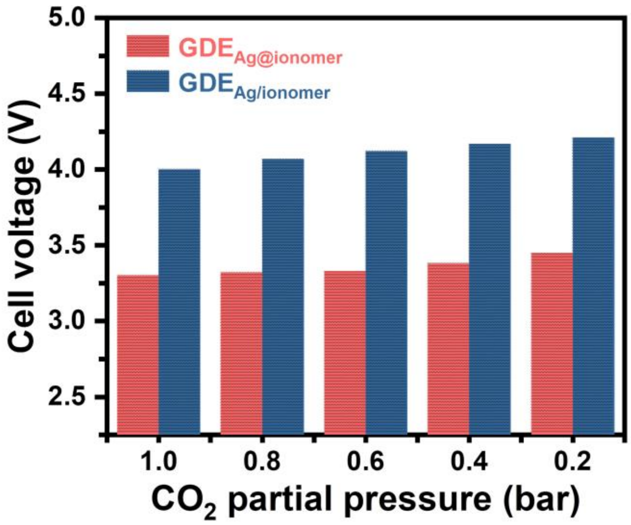

Supplementary Figure 21. Effect of CO_2 partial pressure on the cell voltage of $GDE_{Ag/ionomer}$ and $GDE_{Ag@ionomer}$.

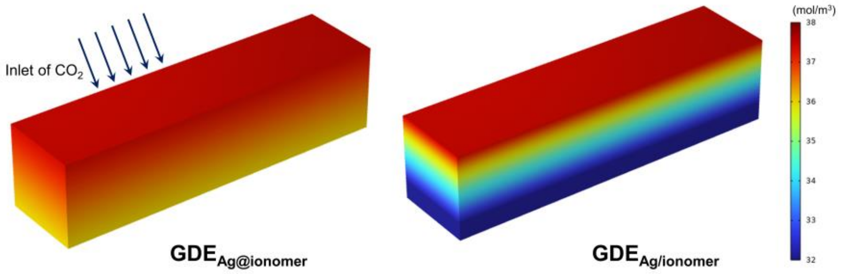

Supplementary Figure 22. Modeled CO_2 feedstock availability along the catalyst layer's surface for $GDE_{Ag@ionomer}$ and $GDE_{Ag/ionomer}$.

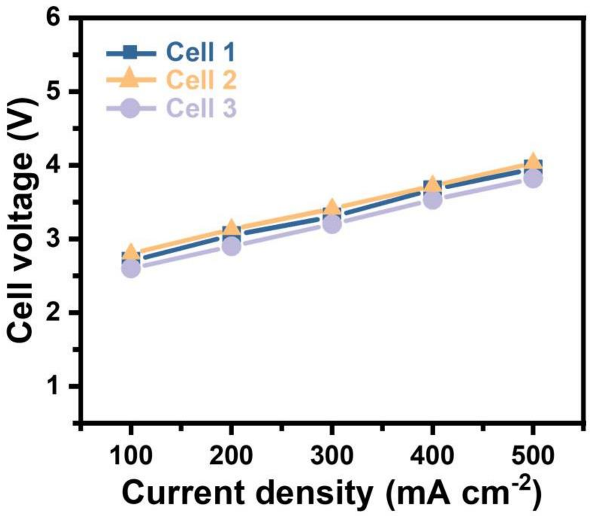

Supplementary Figure 23. Cell voltages of the tandem reactors at different current densities.

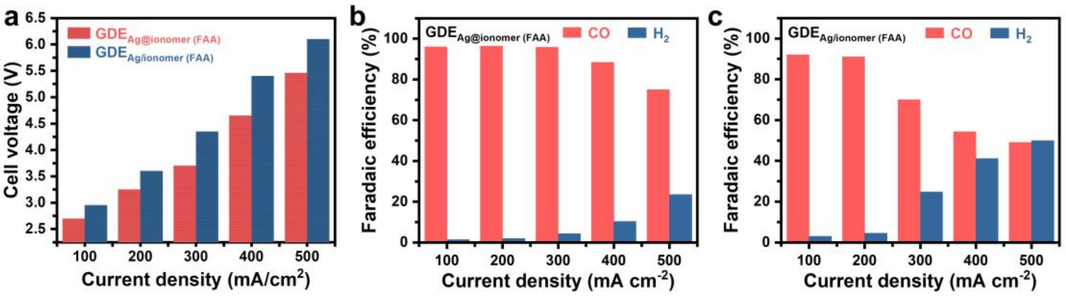

Supplementary Figure 24. (a) Cell voltages of MEAs with $GDE_{Ag@ionomer\ (FAA)}$ and $GDE_{Ag/ionomer\ (FAA)}$ as the GDE at different current densities. FEs for CO and H_2 over (b) $GDE_{Ag@ionomer\ (FAA)}$ and (c) $GDE_{Ag/ionomer\ (FAA)}$ in the MEA.

Supplementary Table 1. Summary of electrochemical properties of Ag@ionomer and Ag/ionomer.

	**Tafel slope ($mV\ dec^{-1}$)**	**Exchange current density ($mA\ cm^{-2}$)**
**Ag@ionomer**	62.9	1.50×10^{-4}
**Ag/ionomer**	60.2	1.42×10^{-4}

Supplementary Table 2. Fitting results of the EIS experiments.

	**$GDE_{Ag@ionomer}$**	**$GDE_{Ag/ionomer}$**
**R_s**	271 mΩ	273 mΩ
**R_{CT}^{Total}**	4.69 Ω	9.59 Ω
**R_d**	2.04 Ω	3.01 Ω
χ^2	0.03	0.04

References

1. Qin Y, Ji X and Jing J *et al.* Size control over spherical silver nanoparticles by ascorbic acid reduction. *Colloids Surf A Physicochem Eng Asp* 2010; **372**: 172-6.
2. Xu Q, Oener SZ and Lindquist G *et al.* Integrated reference electrodes in anion-exchange-membrane electrolyzers: impact of stainless-steel gas-diffusion layers and internal mechanical pressure. *ACS Energy Lett* 2020; **6**: 305-12.
3. Cao H, Pan J and Zhu H *et al.* Interaction regulation between ionomer binder and catalyst: active triple - phase boundary and high performance catalyst layer for anion exchange membrane fuel cells. *Adv Sci* 2021; **8**: 2101744.
4. Wakerley D, Lamaison S and Wicks J *et al.* Gas diffusion electrodes, reactor designs and key metrics of low-temperature CO_2 electrolysers. *Nat Energy* 2022; **7**: 130-43.
5. Lien-Chun W, Alexis T. B and Adam Z. W. Modeling gas-diffusion electrodes for CO_2 reduction. *Phys Chem Chem Phys* 2018; **20**: 16973-84.
